# Increased glymphatic system activity in patients with mild traumatic brain injury

**DOI:** 10.3389/fneur.2023.1148878

**Published:** 2023-05-12

**Authors:** Zhuozhi Dai, Zhiqi Yang, Zhaolin Li, Mu Li, Hongfu Sun, Zerui Zhuang, Weichao Yang, Zehuan Hu, Xiaofeng Chen, Daiying Lin, Xianheng Wu

**Affiliations:** ^1^Department of Radiology, Shantou Central Hospital, Shantou, Guangdong, China; ^2^Department of Radiology, Sun Yat-sen Memorial Hospital, Sun Yat-sen University, Guangzhou, Guangdong, China; ^3^Department of Radiology, Meizhou People's Hospital, Meizhou, Guangdong, China; ^4^Department of Pulmonary and Critical Care Medicine, Sun Yat-sen Memorial Hospital, Sun Yat-sen University, Guangzhou, Guangdong, China; ^5^Department of Neurosurgery, Second Affiliated Hospital of Shantou University Medical College, Shantou, Guangdong, China; ^6^School of Information Technology and Electrical Engineering, University of Queensland, Brisbane, QLD, Australia; ^7^Department of Neurosurgery, Sun Yat-sen Memorial Hospital, Sun Yat-sen University, Guangzhou, Guangdong, China

**Keywords:** mild traumatic brain injury, glymphatic system, human, magnetic resonance imaging, *in vivo*

## Abstract

**Purpose:**

This study aims to investigate the glymphatic system activity changes in patients with mild traumatic brain injury (mTBI), particularly in MRI-negative patients, using analysis along the perivascular space (ALPS) technology.

**Methods:**

A total of 161 mTBI patients (age: 15–92 years old) and 28 healthy controls (age: 15–84 years old) were included in this retrospective study. The mTBI patients were divided into MRI-negative and MRI-positive groups. ALPS index was calculated automatically using whole-brain T1-MPRAGE imaging and diffusion tensor imaging. The Student's *t* and chi-squared tests were performed to compare the ALPS index, age, gender, course of disease, and Glasgow Coma Scale (GCS) score between groups. Correlations among ALPS index, age, course of disease and GCS score were computed using Spearman's correlation analysis.

**Results:**

Increased activity of the glymphatic system was suggested in mTBI patients based on ALPS index analysis, including the MRI-negative patients. There was a significant negative correlation between the ALPS index and age. In addition, a weak positive correlation between the ALPS index and course of disease was also observed. On the contrary, there was no significant correlation between the ALPS index and sex nor between the ALPS index and GCS score.

**Conclusion:**

Our study demonstrated that the activity level of the glymphatic system was enhanced in mTBI patients, even when their brain MRI scans were negative. These findings may provide novel insights for understanding the pathophysiology of mild TBI.

## Introduction

Traumatic brain injury is a major global health problem, with the highest mortality and disability rates among all traumas (1, 2). Moreover, the incidence rate continuously increases yearly. Among them, mild traumatic brain injury (mTBI) accounts for about 80–90% (3). About 420 million patients live with mTBI worldwide yearly (4).

The pathophysiological changes of brain tissue after mTBI have not yet been fully elucidated. MRI as a clinical detection method is important for understanding brain changes after mTBI; however, some patients can be entirely negative on traditional MRI images (5, 6). Recent studies have found that the glymphatic system may be involved in the pathophysiological process of traumatic brain injury (7–9). Altered expression of aquaporin 4 (AQP4), an essential component of the glymphatic system, was found in an animal model of traumatic brain injury (10). In AQP4 gene deletion mice, the glymphatic system dysfunction promotes post-traumatic neuroinflammation and exacerbates cognitive deficits after traumatic brain injury (11). Mounting evidence shows that the changes in glymphatic system function play an important role in the development of mTBI (12, 13), and imaging the glymphatic system in the brain is expected to become a new perspective in the study of mTBI (14, 15). However, evaluating the glymphatic system in human is limited by traditional imaging technologies.

The analysis along the perivascular space (ALPS) method has been proposed in recent years to detect the functional changes of the human glymphatic system non-invasively and indirectly based on diffusion imaging (16, 17). The ALPS refers to the diffusivity along the perivascular space after eliminating the influence of nerve fibers. Specifically, the ALPS index was calculated from the ratio of the diffusivity in three orthogonal directions in the periventricular white matter. Previous studies have demonstrated ALPS index as a potential biomarker for glymphatic system function, with a lower value indicating reduced activity (18, 19). In this study, we aim to investigate the glymphatic system activity changes in mTBI patients, particularly in MRI-negative patients, using the ALPS technology.

## Materials and methods

### Patients

**Figure 1:**
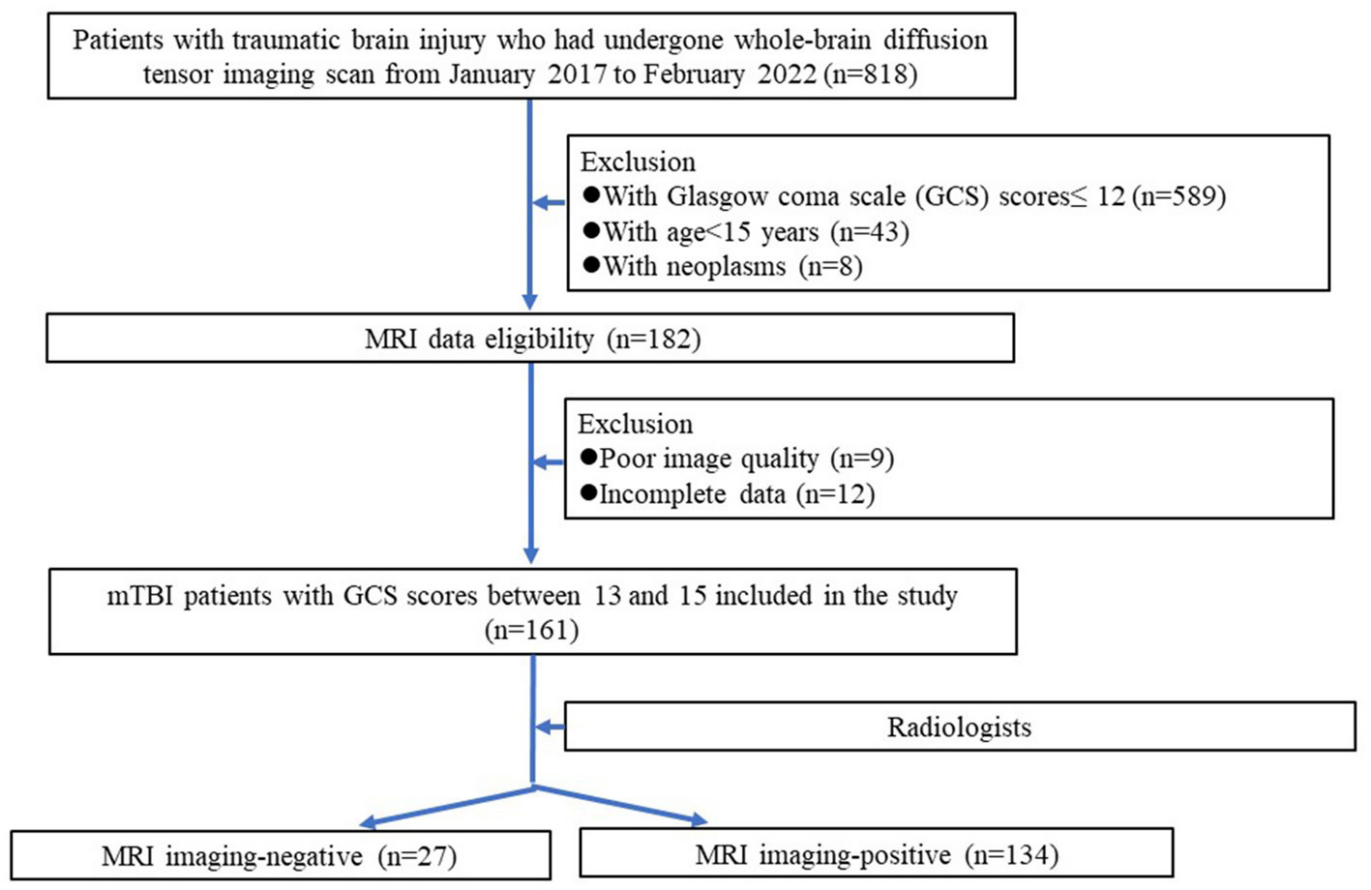
Flowchart of the mTBI patients selection procedure along with the inclusion and exclusion criteria.

Ethical approvals by the institutional review boards were obtained for this retrospective analysis, and the need to obtain informed consent was waived. From January 2017 to February 2022, 814 patients with traumatic brain injury who had undergone whole-brain diffusion tensor imaging (DTI) scans were analyzed retrospectively. The exclusion criteria were as follows: (1) patients with Glasgow coma scale (GCS) scores ≤ 12 (*n* = 589); (2) DTI data incomplete (*n* = 12); (4) patients with neoplasms (*n* = 8); (5) poor DTI image quality (*n* = 9); (6) patients younger than 15 years old (*n* = 43). Finally, 161 mTBI patients with GCS scores between 13 and 15 were included. Among them, 27 patients had negative features on the brain MRI images (namely MRI-negative), and 134 had positive features on the brain MRI images (namely MRI-positive). In addition, another 28 aged-matched healthy subjects without neurological diseases were recruited as controls during the same period. Figure 1 shows the patient recruitment pathway and the inclusion and exclusion criteria.

### MRI acquisition

The MRI data were acquired at a 3.0 T MR scanner (Magnetom Skyra, Siemens), comprising sagittal T1-MPRAGE and whole-brain DTI scans. The patient's head was immobilized with foam pillows inside the head coil to reduce the noise and diminish motion artifacts. DTI images were acquired using a single shot echo planar imaging sequence, with the following parameters: repetition time (TR) = 3,700 ms, echo time (TE) = 95 ms, FOV = 220 × 220 mm, flip angle = 90°, matrix size = 128 × 128 mm^2^, voxel size = 1.7 × 1.7 × 4.0 mm^3^, slice thickness = 4.0 mm, parallel acquisition technique (PAT) = 2, directions = 20, b values of 0 and 1,000 s/mm^2^. The sagittal T1-MPRAGE images were acquired with the following parameters: TR = 2,300 ms, TE = 2.26 ms, TI = 900 ms, FOV = 256 × 256 mm^2^, flip angle = 8°, matrix size = 256 × 256 mm^2^, voxel size = 0.5 × 0.5 × 0.5 mm^3^, slice thickness = 0.50 mm, PAT = 3.

### ALPS analysis

To reduce subjective error, we devised an automated handler of FSL (version 5.0.9) to calculate the ALPS index. The processing flow chart was as follows. First, a glymphatic atlas was built. The Montreal Neurological Institute (MNI) 152 template and JHU-ICBM-Labels atlas were employed as an initial reference frame for alignment. Four 3 mm diameter spherical regions of interest (ROIs) were placed in the bilateral superior longitudinal fascicle (SLF) and superior corona radiata (SCR) according to the previously reported method (16). The MNI coordinates of the centers of the ROIs were set as (64,57,49), (26,57,49), (58,57,49), and (32,57,49). The ROIs were labeled separately and saved as a glymphatic atlas. Second, the individual fractional anisotropy (FA) maps were linearly registered to individual T1 anatomical maps, yielding a transformation matrix. Third, a linear plus non-linear approach was used to morph the individual T1 anatomical maps to MNI space. Fourth, the transformation relationships obtained in steps two and three were combined into a deformation field from the individual FA maps to MNI space. Finally, the deformation field was applied to each diffusion map, and the mean value of the signal was extracted for each ROI in the glymphatic atlas. Average values were taken for identical structures on both sides. The ALPS index was calculated as [(D_xxslf_ + D_xxscr_)/(D_yyscr_ + D_zzslf_)], where D_xxslf_ and D_xxscr_ were the x-axis diffusivity in the areas of SLF and SCR, D_yyscr_ was the y-axis diffusivity in the area of SCR, and D_zzslf_ was the z-axis diffusivity in the area of SLF.

### Statistical analysis

Statistical analyses were performed using R software Version: 3.6.4 (http://www.r-project.org/) and SPSS software version 19 (IBM Corporation). The normality of the variables was evaluated using the Shapiro–Wilk test. The Student's *t* and chi-squared tests were performed to compare the ALPS index, age, gender, course of disease, and GCS score between the mTBI and healthy-control groups. Correlations among ALPS index, age, course of disease and GCS score were computed using Spearman's correlation analysis. Statistical significance was set at *P*-value <0.05 (two-tailed).

## Result

### Patient characteristics

**Figure 2:**
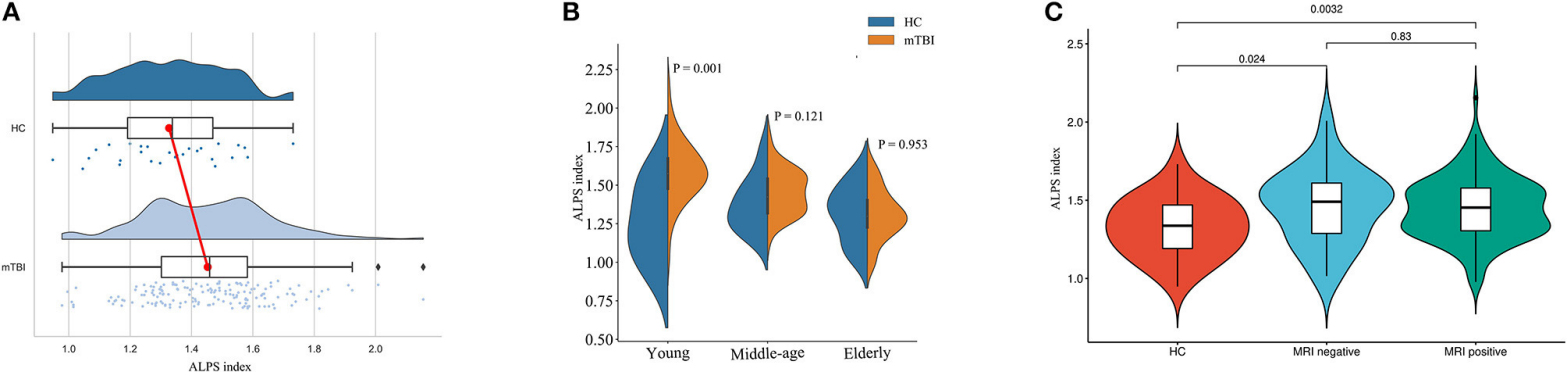
Comparison of ALPS indexes between different subject groups. **(A)** Raincloud plot depicting the ALPS index in each individual. Compared with healthy controls, the mTBI patients have a higher mean ALPS index. **(B)** The data were divided into three subgroups according to age stage, and the differences between mTBI patients and normal controls were compared. The difference was significant in the young group (*P* = 0.001), but not in either the middle-aged or the elderly group. **(C)** The ALPS indexes were higher in both MRI-negative and MRI-positive mTBI patients than in healthy controls (*P* = 0.024 and *P* = 0.003). There was no statistically significant difference in the ALPS index between mTBI patients with MRI-negative and MRI-positive (*P* = 0.830).

A total of 161 mTBI patients aged 15–92 and 28 healthy controls aged 15–84 were included. Patient characteristics of healthy controls (HC) and mTBI groups are compared in Table 1. There was no statistically significant difference in the age between healthy controls and mTBI patients (*P* = 0.097). In contrast, there was a statistically significant difference in the ALPS index between healthy controls and mTBI patients (*P* = 0.003, Figure 2A). Further comparisons showed a significant difference in the young group (*P* = 0.001), but no difference in the middle-aged (*P* = 0.121) and elderly groups (*P* = 0.953) (Figure 2B). Besides, there was a statistically significant difference in sex between healthy controls and mTBI patients (*P* = 0.019).

**Table 1:** Patient characteristics comparison of the healthy controls and mTBI patients.

	**HC (*n* = 28)**	**mTBI (*n* = 161)**	***P***
Age (years)	55.64 ± 17.65	48.88 ± 20.19	0.097^a^
Sex^*^			**0.019** ^b^
Women	13 (46.4%)	40 (24.8%)	
Men	15 (53.6%)	121 (75.2%)	
ALPS index	1.33 ± 0.19	1.45 ± 0.21	**0.003** ^a^
Course of disease	NA	5.55 ± 7.33	NA
GCS score	NA	14.68 ± 1.08	NA

^*^Results are measurements with the corresponding ratios in parentheses, and the remaining results are mean values with standard deviations.

*P*^a^, Student's *t*; *P*^b^, chi-squared test.

ALPS, analysis along the perivascular space; mTBI, mild ttraumatic brain injury; GCS, Glasgow coma scale; NA, not available. The bold values mean *P* < 0.05.

### ALPS index between different subgroups

**Figure 3:**
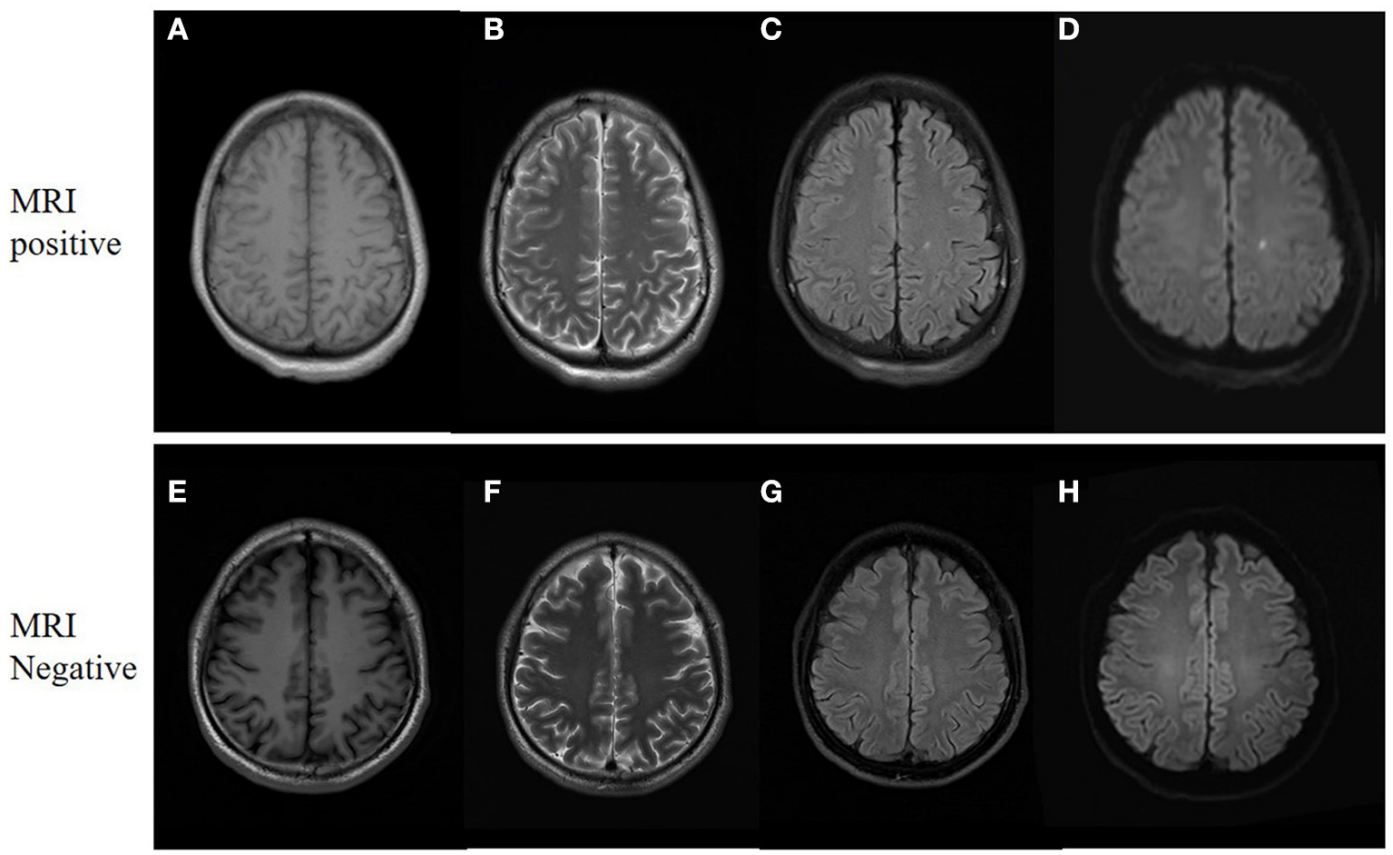
MRI manifestations of two mTBI patients. **(A–D)**, a 31-year-old male with mTBI. Axial non-contrast MRI shows a small punctate contusion of the left corona radiata. The ALPS index is 1.45. **(E–H)**, a 33-year-old male with mTBI. The MRI findings were negative, whereas the ALPS index is 1.46. **(A, E)** are T1 weighted images. **(B, F)** are T2 weighted images. **(C, G)** are T2-Flair images. **(D, H)** are diffusion weighted images.

Of all 161 mTBI patients, 27 (16.7%) were MRI-negative, and 134 (83.2%) were MRI-positive (Table 2). Compared to Healthy controls, both MRI-negative and MRI-positive mTBI sub-groups had significantly higher ALPS indexes (Figure 2C), with *P*-values of 0.023 and 0.003, respectively. The mean ALPS index value of MRI-negative patients was slightly higher than that of MRI-positive patients, but the difference was not statistically significant (*P* = 0.808). Figure 3 illustrates the MRI-negative and MRI-positive manifestations from two mTBI patients with similar ALPS indexes.

**Table 2:** Patient characteristics comparison of the healthy controls and the two mTBI sub-groups.

	**HC (*n* = 28)**	**MRI-negative (*n* = 27)**	***P***	**HC (*n* = 28)**	**MRI-positive (*n* = 134)**	***P***	**MRI-negative (*n* = 27)**	**MRI-positive (*n* = 134)**	***P***
Age	55.64 ± 17.65	40.07 ± 18.18	**0.002**	55.64 ± 17.65	50.65 ± 20.17	0.226	40.07 ± 18.18	50.65 ± 20.17	0.013
Gender^*^			0.059			0.026			0.730
Women	13 (46.4%)	6 (22.2%)		13 (46.4%)	34 (25.4%)		6 (22.2%)	34 (25.4%)	
Men	15 (53.6%)	21 (77.8%)		15 (53.6%)	100 (74.6%)		21 (77.8%)	100 (74.6%)	
Course of disease	NA	5.32 ± 5.07	NA	NA	5.60 ± 7.75	NA	5.32 ± 5.07	5.60 ± 7.75	0.855
GCS scores	NA	14.11 ± 2.26	NA	NA	14.79 ± 0.58	NA	14.11 ± 2.26	14.79 ± 0.58	0.132
ALPS index	1.33 ± 0.19	1.46 ± 0.24	**0.023**	1.33 ± 0.19	1.45 ± 0.20	**0.003**	1.46 ± 0.24	1.45 ± 0.20	0.808

ALPS, analysis along the perivascular space; NA, not available. The bold values mean *P* < 0.05.

### Correlations of ALPS index with age, course of disease, and GCS score

**Figure 4:**
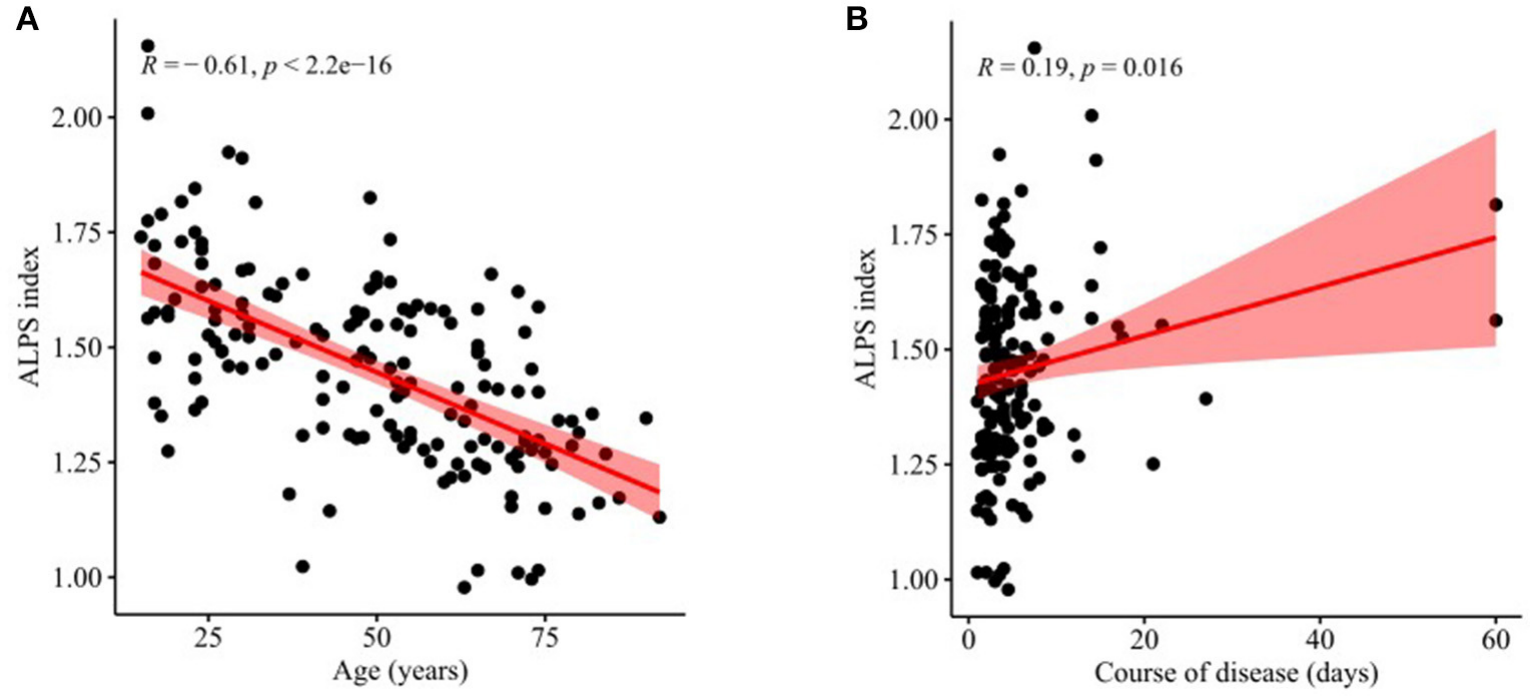
Correlations of ALPS index with age and course of disease. **(A)** There was a significant negative correlation between the ALPS index and age (*r* = −0.61, *P* < 0.001). **(B)** There was a weak positive correlation between the ALPS index and course of disease (*r* = 0.19, *P* = 0.016).

There was a significant negative correlation between the ALPS index and age (*r* = −0.61, *P* < 0.001, Figure 4A) and also a weak positive correlation between the ALPS index and course of disease (*r* = 0.19, *P* = 0.016, Figure 4B). In contrast, there was no significant correlation between the ALPS index and sex (*r* = −0.10, *P* = 0.161) nor between the ALPS index and GCS score (*r* = −0.02, *P* = 0.842).

## Discussion

In this study, we demonstrated that in 161 mTBI patients aged 15–90 s, the activity of the glymphatic system function seemed to be enhanced compared with healthy controls, indicated by the ALPS index. This enhancement was more pronounced at younger ages. Notably, of about seventeen percent of mTBI patients who showed negative brain MRI, changes in the glymphatic system function were detected using the ALPS method. Furthermore, there was a significant negative correlation between the ALPS index and age. A weak positive correlation between the ALPS index and course of disease was also reported. In contrast, there was no significant correlation between the ALPS index and sex nor between the ALPS index and GCS score.

The effect of mTBI on the glymphatic system of the brain is still inconclusive. We found that the activity of the glymphatic system suggested to be increased in patients with mTBI, which might be related to several factors. First, the increased expression of AQP4 after trauma, as previous studies evaluated by western blot or immunohistochemical staining, have been observed in animal models (10, 20, 21). The AQP4, as part of the glymphatic system, has been shown to support perivascular fluid and solute movement (22, 23). In contrast, the dysregulation of AQP4 in gene-knockout mice might inhibit glymphatic system function and exacerbate trauma progression (11). Second, changes in glymphatic system function might be related to hemodynamic fluctuations, as reported in an animal study (24). In a repetitive mTBI model, the glymphatic influx was demonstrated to be increased, whereas the efflux was slower (13). Third, since the glymphatic system plays a crucial role in transporting biomarkers of traumatic brain injury (25, 26), the enhancement might be a compensating mechanism to reduce secondary damage by facilitating the elimination of endotoxic products. It is worth noting that our result was present in mTBI patients, whereas impairment of the glymphatic system could occur in different trauma groups (27–29). Since different studies used trauma of varying severity, and the research objects were focused on young subjects without the elderly, it was difficult to represent the overall population of the disease. The broad age range of patients in this study would better represent the mTBI patient population. Interestingly, our results suggested that the effect of mTBI on the glymphatic system was more pronounced at younger ages, which might be related to the previously published aging of the glymphatic system (30). However, patients with moderate or severe traumatic brain injury may have variable outcomes concerning glymphatic system function.

Another important finding was that changes in the function of the glymphatic system could be detected in patients with negative brain MRIs. Although neuroimaging is an important detection modality for mTBI, many patients still have negative results in standard structural imaging (31, 32). Previous studies have suggested that this is due to the neuropathological changes in these patients being functional impairment rather than structural damage (33). Significant advances in neuroimaging have recently made it possible to study functional abnormalities in mTBI using a variety of methods (34–36). The technology used in this study is based on diffusion imaging non-invasively. Through calculation models to eliminate the interference of fibers in the brain, changes in the diffusivity of the perivascular space can be obtained, representing the activity level of the glymphatic system (16, 17, 37). However, ALPS indexes were conventionally calculated manually, which might introduce subjective error and depend on examiner expertise. To solve the above problem, we proposed an automated processing program in this study to ensure the objectivity of the ALPS index. In contrast, GCS scores are still assessed subjectively and the subjective error might be one of the reasons for the lack of correlation between the two indicators.

As expected, there was a significant negative correlation between the ALPS index and age. According to previous animal experiments, the glymphatic system might be hypo-functioning due to a general decline in cerebrospinal fluid-interstitial fluid exchange in the elder (38–40). This decline was associated with decreased CSF production and arterial pulsatility, which affected glymphatic influx (41–43). In addition, a recent study found that aging mice had impaired meningeal lymphatic function, which might also contribute to glymphatic dysfunction (44). Therefore, the treatment of elderly patients needs to be different from that of younger patients. For elderly patients with mTBI, appropriate treatment to improve cerebral circulation, maintain the activity of the glymphatic system without excessive dehydration, and promote the elimination of endotoxic products might improve the progression of mTBI.

There are several limitations of this study. First, although our cohort was focused on mTBI, there was a substantial difference among etiologies. Therefore, the results may differ from animal models with a single etiology. Second, the sleep status of the study participants was not recorded, which is considered critical for regulating the glymphatic system and deserves analysis in future prospective studies. Third, there was no pathological validation since it was a patient study. Detailed mechanism studies are needed in further experiments.

## Conclusion

Our study demonstrated that the activity level of the glymphatic system was enhanced in mTBI patients, including MRI-negative patients. Moreover, there was a significant negative correlation between the glymphatic system function and age. These findings may provide novel insights for understanding the pathophysiology of mild TBI.

## Data availability statement

The raw data supporting the conclusions of this article will be made available by the authors, without undue reservation.

## Ethics statement

The studies involving human participants were reviewed and approved by Meizhou People's Hospital. Written informed consent from the participants' legal guardian/next of kin was not required to participate in this study in accordance with the national legislation and the institutional requirements.

## Author contributions

ZD, ZY, ZL, ML, and XW: concept and design. ZD, ZY, ZL, ML, HS, ZZ, WY, and ZH: drafting of the manuscript. ZD, ML, HS, XC, DL, and XW: critical revision of the manuscript for important intellectual content. ZY, ZL, HS, and XC: statistical analysis. ZD, ZY, ZL, ML, XC, DL, and XW: administrative, technical, and material support. All authors agreed to be accountable for the content of the work. All authors: acquisition, analysis, and interpretation of data. All authors contributed to the article and approved the submitted version.
